# Reintroducing Consciousness in Psychopathology: Review of the Literature and Conceptual Framework

**DOI:** 10.3389/fpsyg.2020.586284

**Published:** 2020-11-17

**Authors:** Gert Ouwersloot, Jan Derksen, Gerrit Glas

**Affiliations:** ^1^Emergis Psychiatric Hospital, Kloetinge, Netherlands; ^2^Faculty of Social Sciences, Psychology, Radboud University, Nijmegen, Netherlands; ^3^Department of Philosophy, Faculty of Humanities, Free University of Amsterdam, Amsterdam, Netherlands; ^4^Department of Anatomy and Neurosciences, Amsterdam UMC, Faculty of Medicine, Psychiatry, Free University of Amsterdam, Amsterdam, Netherlands

**Keywords:** psychopathology, consciousness, dissociation, transdiagnostic, Jean-Paul Sartre

## Abstract

Alterations in consciousness are among the most common transdiagnostic psychopathological symptoms. Therefore clinical practice would benefit from a clear conceptual framework that guides the recognition, comprehension, and treatment of consciousness disorders. However, contemporary psychopathology lacks such a framework. We describe how pathology of consciousness is currently being addressed in clinical psychology and psychiatry so far, and how the Diagnostic and Statistical Manual of Mental Disorders, Fifth Edition (DSM-5) and International Classification of Diseases, Tenth Edition (ICD-10) refer to this subject. A brief review of the literature on consciousness is then given. After describing psychological perspectives on consciousness and discussing theoretical issues involved in exploring consciousness, we offer a practical clinical working definition of consciousness and we illustrate its connections with a variety of diagnoses. Making use of Jean-Paul Sartre’s distinctions among: states, functions, qualities, and structure, provide a conceptual framework to understand consciousness, to refine diagnostics and to guide the development of therapeutic possibilities in clinical practice.

## Introduction

Many patients with different kinds of psychopathology complain about alterations in their consciousness such as experiencing derealization or depersonalization. For example, they say that the world is “at a distance,” or as if it is observed from behind a screen. Another typical expression is: “I have lost myself.” Many patients say they are confused, alienated, or feel like they are “surviving but not living.”

Ms. C., a middle-aged woman with a moderate-severe depression that persisted for weeks during inpatient treatment, woke up 1 day and experienced a complete recovery from both her affective disorder and her depersonalization. She said, “I am back again. For weeks, I walked, talked, breathed, and ate but I was not really there. It was like being a robot. I know I complained about being depressed, but in fact I did not feel that much. I realized that I still loved my husband but I could not feel the warmth and could not make any connection. But now I am here again.”

According to Edelman and Tononi (2000), “Everyone knows what consciousness is: it is what abandons you every evening when you fall asleep and reappears the next morning when you wake up.” However in spite of what this casual remark suggests, psychology is not at all clear about the meaning of the concept of consciousness (Farthing, 1992). The literature on consciousness often starts with a series of questions: What is it? What are its forms? What does it do? What is its origin? What do we mean by “consciousness”? What is it that, we are trying to understand? Such questions typically end with a comment similar to one made by Chalmers (1995), that “there is nothing that we know more intimately than conscious experience, but there is nothing that is harder to explain.”

In clinical practice, patients complain about problems from a broad range of processes, such as their sense of self, their feelings, memory, hearing voices, visual perception, thinking, concentration, unexplained somatic symptoms, energy, sleep, hypersensitivity, thought intrusions, and so on. Psychopathological symptoms turn out to have a high rate of comorbidity. Caspi et al. (2014) describes evidence pointing to one general underlying dimension that summarizes individuals’ propensity to develop any of the forms of common psychopathologies. Since the formative forces underlying different disorders may be similar, there is a rationale for postulating the potential value of a transdiagnostic approach to psychiatric disorders (Soffer-Dudek et al., 2011; Miller and Rockstroh, 2013; Olatunji et al., 2013; Brodbeck et al., 2014; Sauer-Zavala and Barlow, 2014; Pezzoli et al., 2017; Madeira et al., 2018).

Miss C.’s complaint can be seen as a transdiagnostic symptom referring to a consciousness problem. However, when we use the concept “consciousness” this confronts us with a challenge. Neither the Diagnostic and Statistical Manual of Mental Disorders, Fifth Edition (DSM-5; APA, 2013) nor the International Classification of Diseases, Tenth Edition (ICD-10; WHO, 2010) provides a definition of consciousness, and handbooks of clinical psychology either fail to mention the subject (Stricker et al., 2003; Hersen and Gross, 2008) or they address it very briefly (Sadock, 2000). In Sims’ Symptoms in the Mind, Oyebode (2008) points out that the word “consciousness” is used very freely in psychiatry and that a precise definition is lacking.

In this article, we offer a “working definition” of consciousness that will facilitate the understanding of disorders of consciousness in clinical practice. We give examples of where and how disorders of consciousness show up in clinical practice, being a transdiagnostic phenomenon appearing with a diversity of diagnoses. Our framework aims to link clinical practice with theories in psychology and psychiatry and builds on the work of Sartre. Sartres’ concepts of states, function, quality, and structure of consciousness provide a clearer description and deeper understanding of consciousness disorders, which in turn can lead to improved diagnostic clarity and a considered use of psychotherapeutic possibilities.

## Consciousness in Current Psychopathology

In order to study consciousness legitimately as topic in psychopathology, we must reflect on what is meant by psychopathology. Until recently distress and disability, as operationalization’s of the criterion “harm,” were central in the definition of mental disorder. The DSM-5 changed this focus and gave priority to the concept of dysfunction (Telles-Correia et al., 2018). It defines a mental disorder as a syndrome characterized by a clinically significant disturbance in an individual’s cognition, emotion regulation, or behavior, and reflecting a dysfunction in the psychological, biological, or developmental processes underlying mental functioning. This definition justifies studying consciousness problems as a psychopathological topic because it clearly relates to cognition and psychological processes underlying mental functioning.

The DSM-IV and DSM-5 locate pathology involving consciousness in the group of dissociative disorders (Boon and Draijer, 1995; APA, 2000, 2013; Empty_Memories, 2005; Kihlstrom, 2005; WHO, 2010). In the DSM-5, this group consists of dissociative identity disorder, dissociative amnesia, depersonalization/derealization disorder, other specified dissociative disorder, and unspecified dissociative disorder. All these disorders have dissociative symptomatology as their common denominator. That means there is “… a disruption and/or discontinuity in the normal integration of consciousness, memory, identity, emotion, perception, body representation, motor control, and behavior (APA, 2013).”

The “International Classification of Diseases” (WHO, 2010) does not mention disturbance of consciousness. In the classification of Dissociative (conversion) Disorders (Chapter 5, section F44 of ICD-10), the World Health Organization lists a group of eight dissociative disorders. These are characterized by partial or complete loss of integration of memory, awareness of identity, perception and control of bodily movements. The ICD-10 describes the depersonalization-derealization syndrome in a separate paragraph (F48.1).

Neither the ICD-10, DSM-IV nor DSM-5 provides a definition of consciousness. In the glossary on “Signs and Symptoms in Psychiatry,” Kaplan & Sadock’s Comprehensive Textbook of Psychiatry mentions: “consciousness: State of awareness, with response to external stimuli” (Sadock, 2000, p. 1492).

## From Clinical Phenomenon to Conceptual Thinking About Consciousness

Where do we encounter consciousness and consciousness disturbances in clinical practice, and what are the concepts that we can use to describe and understand them? Clinical phenomena such as self-fragmentation, hallucinations, depersonalization, sense of agency, etc., can in some instances also be accounted for from the perspective of disorders of consciousness (Villagrán, 2003; Holmes et al., 2005). Psychic functions or structures considered to be part of consciousness, such as sense of agency, sense of self, episodic and autobiographic memory, executive functions, perception, insight, monitoring, etc., can be (but not necessarily all are) impaired in patients. Sleep disorders, that in certain cases can be viewed as consciousness disorders, are related to many psychopathological disorders, including depression, bipolar disorder, anxiety disorders, post-traumatic stress disorder (PTSD), obsessive-compulsive disorder, schizophrenia, dissociation, alcoholism, eating disorders, attention deficit hyperactivity disorder, dementia, and autism (Soffer-Dudek, 2017).

We find extensive discussion of the concept of consciousness in the literature on dissociative disorders (Watson, 2001; Brown, 2004; Kihlstrom, 2005; Simeon et al., 2008; Watson et al., 2015). A dimensional line of thought is that in dissociative disorders consciousness can be disturbed more or less severely. According to this theory, consciousness is to be considered a “whole”; and it is a construct that has a dimensional quality. In instances of detachment, disorders of consciousness take the form of varying degrees of losing contact with the self or the environment. Patients use expressions such as: “…feeling ‘spaced out’, ‘unreal’ or … ‘in a dream’…. Patients may describe experiencing events without really feeling they are happening, and that the external world appears lifeless and two-dimensional” (Holmes et al., 2005). This sense of detachment is described on a continuum that indicates the severity of the symptoms and the severity of the functional impairments of the patient. Watson (2001) supports a continuity model of human consciousness and states that people who are prone to interesting, vivid, and unusual experiences during the day also tend to have them at night.

Others believe that consciousness can be categorically or qualitatively disturbed and that compartmentalization takes place (Baker et al., 2003; Holmes et al., 2005). In compartmentalization patients may suffer from a complete inability to steer or control part of their behavior, thinking, or expression. It is characterized by unexplained neurological symptoms such as conversion paralysis, sensory loss, seizures, gait disturbances, and pseudo-hallucinations, as well as other instances of so-called somatoform dissociation (Nijenhuis et al., 1996). Specific symptomatic behavior or thinking anomalies seem to have a psychopathological functionality, but are completely out of reach of the patient’s conscious self-control. This theory accounts for structural alterations in consciousness, fragmentation, and abrupt changes.

Dissociation is often seen in combination with panic disorder, borderline and schizotypal disorders and PTSD. There is also a link between dissociation, a labile sleep-wake cycle, and cognitive problems such as memory errors, problems in attentional control, cognitive failures, and reality testing (Lynn et al., 2012). Experimental and phenomenological research on people with schizophrenia showed that mechanisms like the automatic unconscious processing of sensory information may be impaired, leading to observable abnormalities at a conscious level (Giersch and Mishara, 2017).

These findings highlight the need for a comprehensive formulation of aspects, qualities and functions of consciousness that will improve our understanding of psychopathology.

## Psychological Perspectives on Consciousness

William James’ The Principles of Psychology provides us with valuable concepts for the study of consciousness. James (1842–1910) stated explicitly that psychology cannot be based on basic mental events such as stimuli or simple perceptions and sensations. “The only thing which psychology has a right to postulate at the outset is the fact of thinking itself, and that must first be taken up and analyzed. (…) I use the word thinking (…) for every form of consciousness indiscriminately” (James, 1890). James’ conceptualization is in line with contemporary continental views on psychopathology, where consciousness was the essential mental phenomenon and which was susceptible to transformations caused by mental illness (Dagonet, 1881; Villagrán, 2003).

According to James’ holistic view consciousness is a “whole.” In Chapter 9 of The Principles of Psychology (“The Stream of Thought”), James delineated five characteristics of consciousness:

1. Subjectivity. Consciousness belongs to a unique, independent person.
2. Change. A state of consciousness can never be repeated. Even when exactly the same stimulus is presented the person will have a different experience because of the repetition. Form and content of consciousness change continuously.
3. Continuity. Even when it is interrupted by sleep or anesthesia, people experience consciousness with a certain continuity. Consciousness “before” somehow belongs to consciousness “afterwards.” James’ famous expression “the stream of consciousness” expresses the view that consciousness is normally experienced as a continuous process, regardless of content or state. This experience of continuity is disturbed in dissociative disorders.
4. Intentionality. Consciousness is about something. This intentionality bridges the gap between the conscious person and that which she is conscious of.
5. Selectivity. Consciousness chooses to focus on some stimuli, while neglecting others. We are conscious of only a selection of all the stimuli that are present at each moment.

Phenomenological psychology is grounded in the work of philosophers like Husserl, Heidegger, and Sartre (Husserl, 1950; Sartre, 1990; Mooij, 2006; Giorgi, 2009; Leiviskä Deland et al., 2011; Sass et al., 2011). Consciousness is an important theme in this psychology, together with perception, experience of time, intentionality, the body as “the carrier” of experience, behavior, and social relatedness (Gallagher and Zahavi, 2008). Working in this phenomenological tradition Sass et al. (2011) studied the disturbances of self-experience in schizophrenia. They found that the core abnormality in schizophrenia is a particular kind of disturbance of consciousness and of the sense of self in particular (they use the term “ipseity”). This self or ipseity disturbance has two features that appear to be mutually contradictory, but are in fact complementary. The first is hyper-reflexivity. This refers to a kind of exaggerated self-consciousness, a tendency to direct focal, objectifying attention toward processes and phenomena that would normally be “inhabited” or experienced as part of oneself. The second feature is a diminished self-affection. This refers to a decline in the (passively or automatically) experienced sense of existing as a living and unified subject of awareness. Fuchs describes the interrelatedness of time consciousness, embodiment, and intersubjectivity in mental states and their disturbances in depression and schizophrenia (Fuchs, 2007, 2013). Lanius also points to the experience of time and embodiment, and suggests that time, body, emotion, and thought are the four dimensions that constitute the theory of consciousness (Thompson and Zahavi, 2007; Lanius, 2015). Other examples of the phenomenological study of self and consciousness disorders in schizophrenia are found in the work of Ratcliffe (2008), Stanghellini (2009), Stanghellini and Ballerini (2011), and Lysaker et al. (2015).

Clinical psychologists taking a descriptive approach conceptualize consciousness as something that can be described as an assembly of psychological processes. Consciousness “does” its work with these processes. For instance, perception, feeling, thinking, remembering, and behavior are analyzed, integrated, coordinated, and manipulated in consciousness. Four researchers in this tradition are Ludwig (1966), Tart (1972, 1980), Pekala (1991), and Farthing (1992). They represent the revival of academic psychology’s interest in the phenomenon of consciousness that followed on the “cognitive revolution” that supplanted the period where behaviorism dominated clinical psychology. Although each researcher mentions his own specific assembly of processes, elements of their theories overlap. Table 1 gives a comparative overview of the themes they refer to in studying consciousness.

**Table 1:** Consciousness as an assembly of psychological processes.

Ludwig (1966)	Tart (1969)	Pekala (1991)	Farthing (1992)
Thinking	Information processing	Thinking and rationality	Abstract thinking
Time sense	Sense of time and space	Time sense	Time sense
Self-control		Self-control	Self-control
Emotional expression		Feeling and emotional expression	Emotional expression
Body image		Body image	Body image
Perception	Perception (of external stimuli)	Perception (of external stimuli)	Perception
	Perception (of internal stimuli)	Perception (of internal stimuli)	
		Observation	
Interpretation	Interpretation	Interpretation	Interpretation
Experience of the ineffable			
Sense of age			
Suggestibility			Suggestibility
	Memory	Memory	Memory
			Attention
		Inner speech	Inner speech
		Fantasy	Fantasy
		Level of arousal	Level of arousal
	Feeling of identity	Feeling of identity	Feeling of identity
		Absorption	
	Unconsciousness		
	Emotions		
	Psychomotor function		
	Latent functions		
		Altered states	

All four authors agree that thinking, sense of time, perception, and attribution of meaning are aspects of consciousness. Two or three of them also mention self-control, emotional expression, body sense, suggestibility, inner speech, fantasy, feeling of identity, and level of arousal. Attention, emotions, and psychomotor functions are mentioned once. Ludwig (1966), Tart (1969, 1980), and Farthing (1992) studied altered states of consciousness, such as dreaming, dissociation, drug-induced intoxication, hypnosis, and trance. They pointed out that the quality and balance of psychological functions can change dramatically when states of consciousness alter. Each of the four suggested that assemblies are meaningful and offers interesting starting points for further research.

William James, the phenomenologists and the descriptive psychologists offer a wide array of themes and viewpoints for studying consciousness. Below, we propose a framework that organizes and binds together these diverse elements.

## Exploring Consciousness

One of the reasons that make it difficult to define consciousness is that it has many variations in form and content that are collectively referred to as “altered states of consciousness.” Many of these altered states of consciousness are well-known; for example, sleep, dreams, and daydreams. In addition, consciousness changes during fatigue, is affected by extreme hunger or thirst, and by fever, pain, or serious illness. Altered states of consciousness occur during hypnosis and other forms of religious or concentrative trance, meditation, “peak-experiences” such as being in a flow, focused concentration and in states induced by medication, alcohol, or drugs (Ludwig, 1966; Tart, 1969, 1980; Farthing, 1992). The result of studies of one state of consciousness may differ for another state. For example, it is well-known that depression while under the influence of alcohol can disappear when the person is sober.

Should a description of consciousness be formulated as a statement about the brain or as a statement about the mind? Since Descartes (1641) it seemed obvious that the study of the brain belongs to the domain of the body and the study of mind to the domain of spirit or the psyche. We depend on abstract concepts, language, and thinking for the construction of psychological knowledge, and the gap to the materialistic reality of brain tissue that can be seen, touched, and measured, seems irreconcilable (Derksen, 2012). Ryle (1949) stated that theories about the physical and theories about the mental belong to two different categories. However, developments in “embodied cognition” investigate new ways to approach and overcome the Cartesian gap (Gallagher, 2005; Ratcliffe, 2008; Damasio, 2010; Fuchs, 2013) and also contemporary neuroscience and neurobiology (Koch, 2004) contribute in narrowing the Cartesian mind-body gap. Combining both rigorous phenomenology and rigorous empirical measurement (e.g., Varela’s neurophenomenology) it provides a holistic way to connect the subjective and objective domains of experience (Varela and Shear, 1999). An example is the work of Berkovich-Ohana and her colleagues who studied the underlying neural activity accompanying altered states of consciousness (Berkovich-Ohana et al., 2013). The disciplined introspection of phenomenology connects easily to the Buddhist and Yoga systems as seen in mindfulness and meditation practices. Siegel (2016) combines interpersonal systems theory with neurobiology. His definition of mind incorporates ideas of subtle, non-physical energy systems that relate to Varela’s “radical neurophenomenology” that seeks to observe the process of the arising of thought down to the moment before a subject-object dichotomy arises. When it comes to non-dualistic theories about body and mind and consciousness, one can consult Eastern philosophies and Buddhism (Lutz et al., 2015).

In Daniel Dennett’s view there is no need for subjective experience in the study of consciousness. We capture consciousness, according to Dennett, by describing the integrated total of the cognitive functions that are involved. After that, there is nothing left to explain (Dennett, 2001). This view is elaborated in the neurophilosophical and neuropsychiatric work of Churchland (1989, 1996), who argues that our knowledge about the physical processes in the brain will eventually be able to explain everything we need to know about conscious experience (Damasio, 2010).

Cultural and subcultural factors influence the content and form of psychological phenomena, such as language, perception, behavior, conscience, and thinking (Ornstein, 1972; Farthing, 1992). This also holds true for consciousness (Villagrán, 2003). Therefore it is important to study consciousness in different clinical populations such as people with schizophrenia (Stanghellini, 2004; Stanghellini and Ballerini, 2011; Lysaker et al., 2015), melancholia (Ratcliffe, 2008), or dissociation (Holmes et al., 2005; Kihlstrom, 2005). The study of consciousness in these clinical populations will more than likely result in a different description of consciousness than a study in a group of students or meditating Tibetan monks (Ludwig, 1966).

With respect to research methodology, our presuppositions about consciousness will in the end color the results of the research. Can we study consciousness in an objective or intersubjective way, “from the outside,” gathering empirical facts that can be measured, counted, and weighted? Or must consciousness be approached introspectively, i.e., “from within”? Can consciousness be explored exclusively by an individual who tries to verbalize, comprehend, and understand her own consciousness? These questions are part of the debate on first-person and third person approaches to the study of consciousness (Searle, 1997; Chalmers, 1999; Varela and Shear, 1999; Thompson, 2001; Blackmore, 2003). First-person approaches investigate consciousness (intra)subjectively from a heuristic model of science with research based upon narrativity, phenomenology, and experience (Searle, 1997; Chalmers, 1999; Gallagher and Zahavi, 2008; Giorgi, 2009; Madeira et al., 2018). It assumes that consciousness is accessible exclusively to the individual herself. Only this personal and introspective access to consciousness can be the true source of knowledge about consciousness. Nonetheless, Greenwald and Banaji caution that: “… research that has accumulated since the mid-1980s suggests that … when people attempt to report on their conscious perceptions and judgments, they do so not based on valid introspection but by using traces of past (possibly biased) experience to construct (possibly invalid) theories of current data” (Greenwald and Banaji, 2017). Third-person approaches claim that an objective, empirical study of consciousness is possible. Methods, instruments, and analyses used in this approach fit the empirical logico-positivistic model of science in neuropsychiatry as well as to a medical model way of thinking about psychopathology.

Farthing (1992) writes that in spite of the chain of many impressions and multiple experiences, most people feel that their consciousness is one indivisible whole. Both William James, who said that “Consciousness is an integral thing not made of parts” (James, 1890), and Descartes, who said that “I cannot distinguish in myself any parts, but apprehend myself to be clearly one and entire” (Descartes, 1641), hold such a holistic view of consciousness. On the other hand, neuropsychiatry (Hilgard, 1986) shows how perception, thinking, emotion, and behavior find their origins in the activity of different brain subsystems. Similar to Ludwig (1966), Tart (1980), Pekala (1991), and Farthing (1992), Hilgard conceives consciousness as a compilation of psychological functions. Hilgard’s “Hidden Observer” may be similar to the supervising function commonly associated with consciousness. How can these diverse activities be integrated into a felt whole in consciousness? This is “the binding problem” (Blackmore, 2003). Crick and Koch postulate that the unification in consciousness is accomplished by the synchronization of the activity of brain regions (Crick, 1994; Crick and Koch, 2003). Dissociation disorders illustrate how the whole of consciousness can break down into parts, resulting in symptoms such as amnesia, conversion disorders, fugue states, and alter egos (Putnam, 1995; Holmes et al., 2005; Kihlstrom, 2005).

## Definition of Consciousness: A Proposal

Fully aware of the theoretical pitfalls involved (Blackmore, 2003; Shanon, 2008), we give a working definition of what we mean by consciousness. We propose:

“Consciousness is the total of a human person’s experience consisting of thinking, feeling, perceiving, remembering, fantasizing, dreaming, etc., that is internally represented and where she can communicate about.”

With this definition we confine our understanding of consciousness explicitly to the individual human person. In line with Descartes (1641), James (1890), and Vygotsky (1979), we state that consciousness is “the total” of all mental experiences, which allows for the study of the characteristics of this “total” and its relation to its constituent elements. The formulations “internally represented” and the possibility to “communicate” about it allows for the study of the issues of verbality or non-verbality and the study of embodied experience. We support this definition with a conceptual framework that can cover the psychopathological phenomena, we encounter in clinical practice as well as the ideas and views derived from theories in psychology reviewed above and psychiatry. Our framework resembles the phenomenological matrix presented by Lutz and coworkers that maps styles and stages of mindfulness practice (Lutz et al., 2015). The framework, we describe is derived from writings of Sartre (1936, 1956, 1990) about consciousness using the four concepts of states, functions, qualities, and structure.

### State of Consciousness

Sartre describes consciousness as a psychical phenomenon that can have different states. With the concept “state of consciousness,” he addresses the various specific ways of integration of perception, memory, language, wakefulness, psychomotor ability, and sense of self (or identity). A “state of consciousness” is a “whole” with a characteristic balance, strength, and emphasis in these psychic factors (Tart, 1969, 1980). We encounter psychopathology of the state of consciousness in dissociative disorders, sleep disorders, PTSDs, and conversion disorders, but also in substance-related and addictive disorders. The ease or difficulty in switching between states of consciousness, referring to “suggestibility” and “altered states” (Table 1), and is a measure of William James’ concept of continuity. Soffer-Dudek et al. (2011) describe the interrelatedness of sleep and dream characteristics with psychopathology. They state that a factor expressing unusual dreams and sleep-phenomena can be looked upon as an “altered consciousness trait.” They also found that dissociation and sleep disorders reflect a reciprocal process in which sleep and waking intrude into one another (Soffer-Dudek, 2017). Some sleep problems can be conceived as the outcome of “waking” processes entering sleep. Watson et al. (2015) concluded that sleep disturbances have a broad and little specific relation to psychopathology but with strong links to dissociation and positive symptoms of psychosis and schizotypy (Watson, 2001). A sound sleep is important for a balanced modulation of affects and there is potentially a functional connection between dreaming and recovery from emotional conflict or trauma. On the basis of the neurobiology of sleep, Walker and Van Der Helm outline a model describing the overnight modulation of affective neural systems and the (re)processing of recent emotional experiences, both of which appear to redress the appropriate next-day reactivity of limbic and associated autonomic networks. Where sleep is disrupted the predicted consequences are clinical symptoms like mood disorders (Walker and van Der Helm, 2009).

### Function of Consciousness

Sartre’s concept of “function” expresses the individual’s cognitive operations on the environment. Perception, expression, and self-control (Table 1) are cognitive processes that can be subsumed by this concept. “Function” describes what consciousness enables a person to do. These are processes like intentionality, selectivity, and flexibility. Patients verbalize disruptions in the function of intentionality by saying that they cannot concentrate, cannot focus or cannot feel any interest in things. This is reminiscent of the loss of interest in melancholia or severe depression, the “spacing out or going blank” in dissociation, but also of the aloofness that characterize the schizotaxic disorders or even catatonia in schizophrenia. On the other hand, an overdose of intentionality can focus the patient disproportionately on her surroundings, which can lead to fixations as in the autism spectrum disorders (Nilsson et al., 2019) or to a field dependency such as in a person with a dependent personality disorder. Object orientation, one of the dimensions of the phenomenological matrix described by Lutz et al. (2015), seems to resemble our viewpoint “function.” A disruption in the function of “filtering” or “selectivity” of consciousness leads to patients being overloaded by sensory stimuli, thoughts, and feelings when the filtering function is too loose as in manic disorder, attention disorders, and hyperactivity disorders. It would be interesting to investigate whether a combination of heightened intentionality and selectivity, combined with too little flexibility characterizes the perseveration and tunnel vision so often seen in depression, autism spectrum disorders, and obsessive-compulsive disorders. Where flexibility is disturbed in the sense of there being too much flexibility, we see the shallow floating attention of people with mania. Impairment of the processing of sensory information seems to be a cause of pathology of consciousness in schizophrenia (Giersch and Mishara, 2017). Interestingly this has also been observed in multiple sclerosis, which raises the question whether other pathologies share this consciousness disorder based on difficulty in accessing information. Another kind of disruption of the function of consciousness is in the realm of neurocognitive disorders where, due to brain-related or neurological diseases, the cognitive function deteriorates.

### Quality of Consciousness

The concept “quality of consciousness” indicates how consciousness is narrowed, broadened, or heightened or is clear, concentrated, or clouded (Holmes et al., 2005). Lutz et al. (2015) describe four qualities that (together with three dimensions) constitute the phenomenological matrix of mindfulness practice. These four qualities: aperture, clarity, stability, and effort seem applicable to Sartre’s quality of consciousness. In clinical practice, we hear patients say that they feel fuzzy, “in a haze” or “like a zombie.” People with depression often exhibit a specific cognitive bias, paying more attention to negative information and negative memories (Gaddy and Ingram, 2014). Quality of consciousness relates to psychological processes such as “level of arousal,” “sense of time,” and “fantasy” (Table 1).

We can also refer to Janet’s concept of “psychological tension” (Ellenberger, 1970; van der Kolk and van der Hart, 1989). Human functioning, according to Janet, can be viewed as the result of the integration of perception and behavior. It consists of several levels which form a hierarchy. At the highest level, which is also the level most difficult to reach and to retain, functioning is focused in the present and voluntary action and attention are synthesized. Janet’s concept of “psychological tension” describes the quality of human functioning with respect to this synthesis. It refers to the vitality, psychological equilibrium and range of attention in humans (Masek, 1989). At the highest synthesized level, which Janet calls fonction du réel, consciousness is clear, alert, broad, and adequately attuned both to reality and to emotional and cognitive aspects of the individual. At the next level of synthesis, people function chiefly in a daily and habitual way, as a routine, without much interest or focused attention. At this second level as well as at lower levels, only a small part of the interaction between the individual and his/her environment requires conscious awareness, according to Janet (van der Kolk and van der Hart, 1989). At the third level, we find imagination, fantasy, and daydreaming. Interestingly, Janet also localizes abstract reasoning at this level. Abstract thought can be valued as an aspect of higher intelligence but can also be seen as dysfunctional and as blocking emotional growth. Next, Janet discerns the level of emotional reactions which, in his view, is inferior to the higher levels of imagination and habitual behavior. At the fifth and lowest level of synthesis, we find uncoordinated spontaneous muscular movements and reflexes. In Janet’s view, psychopathology is connected with the deterioration of the synthesizing function of voluntary action and attention, leading to a lowering of psychological tension i.e., a lowering of vitality. This lowering can occur as a result of somatic problems, diseases, or psychological conflicts or trauma (Ellenberger, 1970). Janet’s theory has proved to be useful in understanding the etiology of dissociative disorders resulting from actual trauma (van der Kolk and van der Hart, 1989). Freudian psychoanalytic theory points to the serial and logical qualities of consciousness and its orientation toward reality. A breakdown of these qualities results in disruptive, impulse-control and conduct disorders or to the chaotic and bizarre psychotic symptoms of schizophrenia. At the other extreme, excessive focus on reality and logic is one of the distinctive aspects of Asperger syndrome.

### Structure of Consciousness

In Sartre’s view, the concept of the structure of consciousness denotes the relation of the individual to his/her surroundings. In the structure of consciousness, he discerns three positions. The first position is where the person behaves without thinking. The individual acts in an immersed or automatic way and Sartre calls this “the non-reflexive consciousness.” It corresponds to the kind of “pre-reflexive self-awareness,” Raffman describes which is a nonconceptual and thinner awareness of one’s own conscious mental states that goes without cognitive achievement, without judgment, without conceptual thinking. One is simply aware (Raffman, 1999). This level corresponds to the first level of consciousness (i.e., minimal consciousness) described in studies of the development of consciousness in infants (Zelazo et al., 2007). It also reflects what happens at Janets second and lower levels of consciousness where individuals lose “psychological tension.” The second position is where individuals act and think about their acting. This is called “reflexive consciousness.” The third position is characterized as “positional consciousness” where individuals not only act and think about their acting, but also think about their thinking. This reflects meta-awareness and a high level of dereification (Lutz et al., 2015). According to Raffman in Autism Spectrum Disorders only the second and third levels are disturbed, whereas the first-person access to the pre-reflective self-awareness is unaffected. In Table 1, psychological processes such as “interpretation,” “thinking,” and “feeling of identity” are identified as examples of structures of consciousness.

## Disorders of Consciousness and Psychotherapy

The last few decades have witnessed the development of psychotherapeutic strategies that explicitly intervene on aspects of the patients’ consciousness. We see this in Mentalization Based Treatment (Fonagy et al., 2002; Bateman and Fonagy, 2004), Cognitive Psychotherapy (Teasdale et al., 1995), Acceptance and Commitment Therapy (Hayes, 2004), Mindfulness (Kabat-Zinn, 2003, 2005; Lanius, 2015), Psychodynamics (Fonagy et al., 2002; Westen et al., 2007), and in the experimental psychopathology of attentional processes. Lanius (2015) suggests that pathologies of consciousness such as dissociative flashbacks may successfully be treated by strengthening the sense of self by applying present-centerd psychotherapies in combination with exposure-based treatments.

We think that one of the goals of psychotherapy is to reconstruct psychological architecture by means of activating primary emotions in the here-and-now and changing consciousness by verbalizing the experiences (Derksen, 2012). Janet thought that the quality of consciousness can be trained by mastering a series of problems of successive complexity and duration. The exploration of attention processes and aspects of consciousness have increased our understanding of and provided psychotherapeutic tools for the treatment of psychopathology (Teasdale et al., 1995; Kabat-Zinn, 2003, 2005; Bateman and Fonagy, 2004; Hayes, 2004).

With the concepts of states, function, quality, and structure of consciousness, together with William James’ characteristics of consciousness identity, continuity, change, intentionality, and selectivity, we offer tools for understanding disorders of consciousness in clinical psychopathology. They specify the diagnostics of consciousness disorders and provide logical and comprehensive direction to therapeutic interventions. This can involve strengthening intentionality in withdrawn or dissociated patients by helping them to focus on their surrounding reality, or can involve reversing the attention of people with a sleep disorder from their surrounding back to their emotions and inner thoughts. Psychotherapy focusing on consciousness can involve strengthening the filter of perception in overstimulated people with mania or ADHD or widening perception where depression or OCD traps people in fixated perseverations or narrowed tunnel vision. Intervening on consciousness can involve addressing the lack of attuned relation to others and the world in ASD (Nilsson et al., 2019), enhancing self-image, exploring identity, or clarifying self-perception and can also be about focusing on the self-disturbance in schizophrenia-spectrum disorders where patients can experience overwhelming anxiety when they feel their identity dissolving (Stanghellini, 2004; Sass et al., 2011; Stanghellini and Ballerini, 2011; Lysaker et al., 2015).

## Discussion: Consciousness Exists

We focused on reintroducing the concept of consciousness to psychopathology because disorders of consciousness are among the most frequent transdiagnostic symptoms. Psychodiagnostics and psychotherapy will benefit from the development of a conceptual framework that integrates facts and theories of consciousness that we find in both the scientific literature and in clinical practice. The framework, we propose describes consciousness with the concepts of states, qualities, functions, and structure proposed by Sartre. They are sufficiently comprehensive to be capable of encompassing a broad variety of clinical symptoms as well as the diverse clinical and scientific theories of consciousness and its disorders. Future research will have to clarify the validity, usefulness, and applicability of the framework for understanding psychopathology and give direction to psychotherapeutic strategy. The research agenda will also point to the study of the relations of aspects of the framework of consciousness with physiological and behavioral markers and the neuropsychiatric findings. For instance in needs to be clarified how different states of consciousness are reflected in the activities of the central-executive network, default-mode network, and the salience network (Carhart-Harris and Friston, 2010; Goulden et al., 2014; Lutz et al., 2015). And to constitute the empirical validity of the model data must be collected from first- and second-person perspectives using phenomenological inquiry, psychometric tests, behavioral observation, and neurophysiological scans and measurements to certify the presented concepts in different populations and different cultures.

For now, many questions remain. For instance, how can we address disorders of consciousness in the light of identity disturbance, in depression, or anxiety disorders, in personality disorders? What will be the most effective diagnostic method? What kinds of consciousness disorders can we agree upon and how will they be related to the DSM-5 and ICD-10 concepts? How can we treat disorders of consciousness? What are the developmental processes in consciousness and is there a way to advance them? There is a great need for research in this area that gives us sound experimental proof and evidence based knowledge. However, a concept like consciousness seems to steer psychological science away from the quantitative, experimental, and naturalistic sciences and toward the heuristic, phenomenological, and qualitative humanities. Is this the direction psychology will move?

Behind the questions formulated at the level of clinical science, more profound questions about the concept of consciousness remain on the level of theory of science. For example, how does consciousness relate to the body-mind problem? Can consciousness be studied objectively or intersubjectively with empirical methods or can knowledge about consciousness only be gathered “from within” with the introspective method (Boer et al., 2008)? Is consciousness an accompanying quality of mental processes or is it a mental process in itself?

More than a century after James (1904) asked the question “Does consciousness exist?,” we can answer: “Yes, consciousness exists in the way that patients, no matter their diagnosis, demonstrate symptoms that productively can be studied with the transdiagnostic concept of consciousness.” Our patients tell us that consciousness exists and it is an important subject to focus on every patient like Miss C. mentioned at the beginning of this article suffers a psychopathology that goes with a specific constellation of the state, function, quality, and structural features of her consciousness. We think it is time to call attention to the subject of consciousness and to explore the relation of the disorders of consciousness to psychopathology.

## Author Contributions

GO is principal author who is responsible for the ideas and vision of the article. JD and GG read the manuscript, commented on it, and stimulated the development of the article by providing feedback and suggesting viewpoints. All authors contributed to the article and approved the submitted version.

### Conflict of Interest

The authors declare that the research was conducted in the absence of any commercial or financial relationships that could be construed as a potential conflict of interest.

## References

[ref1] APA (2000). Diagnostic and statistical manual of mental disorders: DSM-IV-TR. Arlington, VA: American Psychiatric Publishing, Inc.

[ref2] APA (2013). Diagnostic and statistical manual of mental disorders. 5th Edn. Arlington, VA: American Psychiatric Association.

[ref3] Baker D. Hunter E. Lawrence E. Medford N. Patel M. Senior C. (2003). Depersonalisation disorder: clinical features of 204 cases. Br. J. Psychiatry 182, 428–433. 10.1192/bjp.182.5.428 12724246

[ref4] Bateman A. Fonagy P. (2004). Psychotherapy for borderline personality disorder: Mentalization based treatment. New York: Oxford University Press.

[ref5] Berkovich-Ohana A. Dor-Ziderman Y. Glicksohn J. Goldstein A. (2013). Alterations in the sense of time, space, and body in the mindfulness-trained brain: a neurophenomenologically-guided MEG study. Front. Psychol. 4:912. 10.3389/fpsyg.2013.00912, PMID: 24348455 PMC3847819

[ref6] Blackmore S. (2003). Consciousness, an introduction. London: Hodder & Stoughton.

[ref7] Boer J. A. D. Reinders A. A. T. S. Glas G. (2008). Special section: on looking inward: revisiting the role of introspection in neuroscientific and psychiatric research. Theory Psychol. 18, 380–403. 10.1177/0959354308089791

[ref8] Boon S. Draijer N. (1995). Screening en Diagnostiek van Dissociatieve Stoornissen (Screening and diagnosing dissociative disorders; original in Dutch). Lisse: Sets & Zeitlinger.

[ref9] Brodbeck J. Stulz N. Itten S. Regli D. Znoj H. Caspar F. (2014). The structure of psychopathological symptoms and the associations with DSM-diagnoses in treatment seeking individuals. Compr. Psychiatry 55, 714–726. 10.1016/j.comppsych.2013.11.001, PMID: 24360603

[ref10] Brown R. J. (2004). Psychological mechanisms of medically unexplained symptoms: an integrative conceptual model. Psychol. Bull. 130, 793–812. 10.1037/0033-2909.130.5.793, PMID: 15367081

[ref11] Carhart-Harris R. L. Friston K. J. (2010). The default-mode, ego-functions and free-energy: a neurobiological account of Freudian ideas. Brain 133, 1265–1283. 10.1093/brain/awq010, PMID: 20194141 PMC2850580

[ref12] Caspi A. Houts R. M. Belsky D. W. Goldman-Mellor S. J. Harrington H. Israel S.. (2014). The p factor: one general psychopathology factor in the structure of psychiatric disorders? Clin. Psychol. Sci. 2, 119–137. 10.1177/2167702613497473, PMID: 25360393 PMC4209412

[ref13] Chalmers D. J. (1995). Facing up to the problem of consciousness. J. Conscious. Stud. 3, 200–219.

[ref14] Chalmers D. J. (1999). First-person methods in the science of consciousness [Online]. Available at: http://consc.net/papers/firstperson.html (Accessed Fall, 2020).

[ref15] Churchland P. S. (1989). Neurophilosophy: Toward a unified science of the mind-brain. Bradford Massachusetts: The MIT press.

[ref16] Churchland P. S. (1996). The hornswoggle problem. J. Conscious. Stud. 3, 402–408.

[ref17] Crick F. (1994). The astonishing hypothesis. New York: Scribner.

[ref18] Crick F. Koch C. (2003). A framework for consciousness. Nat. Neurosci. 6, 119–126. 10.1038/nn0203-119, PMID: 12555104

[ref19] Dagonet H. (1881). Conscience et aliénation mentale. Ann. Med. Psychol. 5:29.

[ref20] Damasio A. (2010). Self comes to mind. Constructing the conscious brain. London: William Heinemann.

[ref21] Dennett D. (2001). Are we explaining consciousness yet? Cognition 79, 221–237. 10.1016/S0010-0277(00)00130-X, PMID: 11164029

[ref22] Derksen J. (2012). Bevrijd de psychologie (Liberate psychology from the myth of mind science). Amsterdam: Bert Bakker.

[ref23] Descartes R. (1641). Meditations on first philosophy. With selections from the objections and replies. Cambridge: Cambridge University Press.

[ref24] Edelman G. M. Tononi G. (2000). Consciousness. How matter becomes imagination. London: Penguin Books.

[ref25] Ellenberger H. F. (1970). The discovery of the unconscious: The history and evolution of dynamic psychiatry. London: Allen Lane.

[ref26] Empty_Memories (2005). Dissociatieve Stoornissen (Dissociative Disorders) [Online]. Available at: http://www.ememo.nl/disspectrum.html (Accessed December 12, 2005).

[ref27] Farthing G. W. (1992). The psychology of consciousness. Englewood Cliffs, NJ: Prentice Hall.

[ref28] Fonagy P. Gergely G. Jurist E. Target M. (2002). Affect regulation, mentalization and the development of the self. New York: Other Press.

[ref29] Fuchs T. (2007). The temporal structure of intentionality and its disturbance in schizophrenia. Psychopathology 40, 229–235. 10.1159/000101365, PMID: 17396049

[ref30] Fuchs T. (2013). Temporality and psychopathology. Phenomenol. Cogn. Sci. 12, 75–104. 10.1007/s11097-010-9189-4

[ref31] Gaddy M. A. Ingram R. E. (2014). A meta-analytic review of mood-congruent implicit memory in depressed mood. Clin. Psychol. Rev. 34, 402–416. 10.1016/j.cpr.2014.06.001, PMID: 24980699

[ref32] Gallagher S. (2005). How the body shapes the mind. Oxford: Clarendon Press.

[ref33] Gallagher S. Zahavi D. (2008). The phenomenological mind. New York: Routledge.

[ref34] Giersch A. Mishara A. L. (2017). Is schizophrenia a disorder of consciousness? Experimental and phenomenological support for anomalous unconscious processing. Front. Psychol. 8:1659. 10.3389/fpsyg.2017.01659, PMID: 29033868 PMC5625017

[ref35] Giorgi A. (2009). The descriptive phenomenological method in psychology: A modified Husserlian approach. Pittsburgh, Pennsylvania: Duquesne University Press.

[ref36] Goulden N. Khusnulina A. Davis N. J. Bracewell R. M. Bokde A. L. McNulty J. P.. (2014). The salience network is responsible for switching between the default mode network and the central executive network: replication from DCM. NeuroImage 99, 180–190. 10.1016/j.neuroimage.2014.05.052, PMID: 24862074

[ref37] Greenwald A. G. Banaji M. R. (2017). The implicit revolution: reconceiving the relation between conscious and unconscious. Am. Psychol. 72, 861–871. 10.1037/amp0000238, PMID: 29283625

[ref38] Hayes S. C. (2004). Mindfulness and acceptance: Expanding the cognitive-behavioral tradition. New York: The Guilford Press.

[ref39] Hersen M. Gross A. M. (2008). Handbook of clinical psychology. Hoboken, New Jersey: John Wiley & Sons.

[ref40] Hilgard E. R. (1986). Divided consciousness: Multiple controls in human thought and action. New York: Wiley.

[ref41] Holmes E. A. Brown R. J. Mansell W. Fearon R. P. Hunter E. C. M. Frasquilho F.. (2005). Are there two qualitatively distinct forms of dissociation? A review and some clinical implications. Clin. Psychol. Rev. 25, 1–23. 10.1016/j.cpr.2004.08.006, PMID: 15596078

[ref42] Husserl E. (1950). Cartesianische Meditationen und Pariser Vortraege. Den Haag, Netherlands: M. Nijhoff.

[ref43] James W. (1890). The principles of psychology. London: MacMillan.

[ref44] James W. (1904). Does consciousness exist? J. Philos. Psychol. Sci. Methods 1:14.

[ref45] Kabat-Zinn J. (2003). Mindfulness-based interventions in context: past, present, and future. Clin. Psychol. Sci. Pract. 10, 144–156. 10.1093/clipsy.bpg016

[ref46] Kabat-Zinn J. (2005). Coming to our senses: Healing ourselves and the world through mindfulness. New York: Hyperion.

[ref47] Kihlstrom J. F. (2005). Dissociative disorders. Annu. Rev. Clin. Psychol. 1, 227–253. 10.1146/annurev.clinpsy.1.102803.143925 17716088

[ref48] Koch C. (2004). The quest for consciousness. A neurobiological approach. Englewood (Colorado): Roberts and Company Publishers.

[ref49] Lanius R. A. (2015). Trauma-related dissociation and altered states of consciousness: a call for clinical, treatment, and neuroscience research. Eur. J. Psychotraumatol. 6:27905. 10.3402/ejpt.v6.27905, PMID: 25994026 PMC4439425

[ref50] Leiviskä Deland A. C. Karlsson G. Fatouros-Bergman H. (2011). A phenomenological analysis of the psychotic experience. Hum. Stud. 34, 23–42. 10.1007/s10746-011-9174-0

[ref51] Ludwig A. M. (1966). Altered states of consciousness. Arch. Gen. Psychiatry 15, 225–234. 10.1001/archpsyc.1966.01730150001001, PMID: 5330058

[ref52] Lutz A. Jha A. P. Dunne J. D. Saron C. D. (2015). Investigating the phenomenological matrix of mindfulness-related practices from a neurocognitive perspective. Am. Psychol. 70, 632–658. 10.1037/a0039585, PMID: 26436313 PMC4608430

[ref53] Lynn S. J. Lilienfeld S. O. Merckelbach H. Giesbrecht T. van der Kloet D. (2012). Dissociation and dissociative disorders: challenging conventional wisdom. Curr. Dir. Psychol. Sci. 21, 48–53. 10.1177/0963721411429457

[ref54] Lysaker P. H. Vohs J. Minor K. S. Irarrázaval L. Leonhardt B. Hamm J. A.. (2015). Metacognitive deficits in schizophrenia: presence and associations with psychosocial outcomes. J. Nerv. Ment. Dis. 203, 530–536. 10.1097/NMD.0000000000000323, PMID: 26121151

[ref55] Madeira L. Filipe T. Cavaco T. Pienkos E. Figueira M. L. (2018). The loss of nosological validity: why and how should we consider disturbances of subjective world experience? Psicopatologia Fenomenológica Contemporânea 17, 29–46. 10.37067/rpfc.v7i2.970

[ref56] Masek R. (1989). The overlooked problem of consciousness in psychoanalysis: Pierre Janet revisited. Humanist. Psychol. 17, 274–279. 10.1080/08873267.1989.9976859

[ref57] Miller G. A. Rockstroh B. (2013). Endophenotypes in psychopathology research: where do we stand? Annu. Rev. Clin. Psychol. 9, 177–213. 10.1146/annurev-clinpsy-050212-185540, PMID: 23297790

[ref58] Mooij A. W. M. (2006). De psychische realiteit: Psychiatrie als geesteswetenschap. (psychic reality: Psychiatry as science of the mind original in Dutch). Amsterdam: Boom.

[ref59] Nijenhuis E. R. S. D. Spinhoven P. P. D. Van Dyck R. M. D. P. D. Der Hart O. V. P. D. Vanderlinden J. P. D. (1996). The development and psychometric characteristics of the somatoform dissociation questionnaire (SDQ-20). J. Nerv. Ment. Dis. 184, 688–694. 10.1097/00005053-199611000-00006, PMID: 8955682

[ref60] Nilsson M. Handest P. Nylander L. Pedersen L. Carlsson J. Arnfred S. (2019). Arguments for a phenomenologically informed clinical approach to autism spectrum disorder. Psychopathology 52, 153–160. 10.1159/000500294, PMID: 31170725

[ref61] Olatunji B. O. Naragon-Gainey K. Wolitzky-Taylor K. B. (2013). Specificity of rumination in anxiety and depression: a multimodal meta-analysis. Clin. Psychol. Sci. Pract. 20, 225–257. 10.1111/cpsp.12037

[ref62] Ornstein R. E. (1972). The psychology of consciousness. San Fransisco: Freeman and Company.

[ref63] Oyebode F. (2008). Sim’s symptoms in the mind. An introduction to descriptive psychopathology. Edinburgh: Saunders Elsevier.

[ref64] Pekala R. J. (1991). Quantifying consciousness. San Francisco: Freeman and Company.

[ref65] Pezzoli P. Antfolk J. Santtila P. (2017). Phenotypic factor analysis of psychopathology reveals a new body-related transdiagnostic factor. PLoS One 12:e0177674. 10.1371/journal.pone.0177674, PMID: 28542328 PMC5436748

[ref66] Putnam F. W. (1995). “Development of dissociative disorders” in Developmental psychopathology: Theory and methods. eds. Cicchetti D. Cohen D. (New York: Wiley).

[ref67] Raffman D. (1999). What autism may tell us about self-awareness: a commentary on Frith and Happé. Mind Lang. 14, 23–31. 10.1111/1468-0017.00101

[ref68] Ratcliffe M. (2008). Feelings of Being. Oxford: University Press.

[ref69] Ryle G. (1949). The concept of mind. London: Hutchinson & Co, Ltd.

[ref70] Sadock B. J. (2000). Kaplan & Sadock’s comprehensive textbook of psychiatry (2 volume set). Philadelphia: Lippincott Williams & Wilkins.

[ref71] Sartre J. P. (1936). “La Transcendance de l’Ego” in Recherches philosophiques. Paris: J.Vrin.

[ref72] Sartre J. P. (1956). Being and nothingness. New York: Philosophical Library.

[ref73] Sartre J. P. (1990). The transcendence of the ego. New York: Hill and Wang.

[ref74] Sass L. Parnas J. Zahavi D. (2011). Phenomenological psychopathology and schizophrenia: contemporary approaches and misunderstandings. Philos. Psychiatry Psychol. 18, 1–23. 10.1353/ppp.2011.0008

[ref75] Sauer-Zavala S. Barlow D. H. (2014). The case for borderline personality disorder as an emotional disorder: implications for treatment. Clin. Psychol. Sci. Pract. 21, 118–138. 10.1111/cpsp.12063

[ref76] Searle J. (1997). The mystery of consciousness. New York: New York Review of Books.

[ref77] Shanon B. (2008). A psychological theory of consciousness. J. Conscious. Stud. 15:42.

[ref78] Siegel D. J. (2016). Mind: A journey to the heart of being human (Norton series on interpersonal neurobiology). New York and London: WW Norton & Company.

[ref79] Simeon D. Kozin D. S. Segal K. Lerch B. Dujour R. Giesbrecht T. (2008). De-constructing depersonalization: further evidence for symptom clusters. Psychiatry Res. 157, 303–306. 10.1016/j.psychres.2007.07.007, PMID: 17959254

[ref80] Soffer-Dudek N. (2017). Arousal in nocturnal consciousness: how dream- and sleep-experiences may inform us of poor sleep quality, stress, and psychopathology. Front. Psychol. 8:733. 10.3389/fpsyg.2017.00733, PMID: 28539902 PMC5423938

[ref81] Soffer-Dudek N. Shalev H. Shiber A. Shahar G. (2011). Role of severe psychopathology in sleep-related experiences: a pilot study. Dreaming 21, 148–156. 10.1037/a0022865

[ref82] Stanghellini G. (2004). Disembodied spirits and Deanimated bodies. The psychopathology of common sense. Oxford: Oxford University Press.

[ref83] Stanghellini G. (2009). The meanings of psychopathology. Curr. Opin. Psychiatr. 22, 559–564. 10.1097/YCO.0b013e3283318e36, PMID: 19734787

[ref84] Stanghellini G. Ballerini M. (2011). What is it like to be a person with schizophrenia in the social world? A first-person perspective study on schizophrenic dissociality – part 1: state of the art. Psychopathology 44, 172–182. 10.1159/000322637, PMID: 21412031

[ref85] Stricker G. Widiger T. A. Weiner I. B. (2003). Handbook of psychology: Volume 8, clinical psychology. Hoboken: John Wiley & Sons.

[ref86] Tart C. T. (1969). Altered states of consciousness. New York: Wiley & Sons, Inc.

[ref87] Tart C. T. (1972). States of consciousness and state-specific sciences. Science 176, 1203–1210. 10.1126/science.176.4040.1203, PMID: 17790404

[ref88] Tart C. T. (1980). “A systems approach to altered states of Consciousness” in The psychobiology of consciousness. eds. Davidson J. M. Davidson R. J. (New York: Plenum Press), 243–270.

[ref89] Teasdale J. D. Segal S. V. Williams J. M. G. (1995). How does cognitive therapy prevent relapse and why should attentional control (mindfulness) training help? Behav. Res. Ther. 33, 225–239. 10.1016/0005-7967(94)e0011-7 7872934

[ref90] Telles-Correia D. Saraiva S. Gonçalves J. (2018). Mental disorder—the need for an accurate definition. Front. Psychol. 9:64. 10.3389/fpsyt.2018.00064, PMID: 29593578 PMC5857571

[ref91] Thompson E. (2001). Between ourselves: Second-person issues in the study of consciousness; a special issue of the journal of consciousness studies. Thorverton, Devon: Imprint Academic.

[ref92] Thompson E. Zahavi D. (2007). “Philosophical issues: phenomenology” in Cambridge handbook of consciousness studies. eds. Zelazo M. M. P. D. Thompson E. (Cambridge University Press: New York), 67–87.

[ref93] van der Kolk B. A. van der Hart O. (1989). Pierre Janet and the breakdown of adaptation. Am. J. Psychiatr. 146, 1530–1540. 10.1176/ajp.146.12.1530, PMID: 2686473

[ref94] Varela F. J. Shear J. (1999). The view from within: First person approaches to the study of consciousness. A special issue of the journal of consciousness studies. Thorverton, Devon: Imprint Academic.

[ref95] Villagrán J. M. (2003). Consciousness disorders in schizophrenia: a forgotten land for psychopathology. Int. J. Psychol. Psychol. Ther. 3, 209–234.

[ref96] Vygotsky L. S. (1979). Consciousness as a problem in the psychology of behavior. Sov. Psychol. 17, 3–35.

[ref97] Walker M. P. van Der Helm E. (2009). Overnight therapy? The role of sleep in emotional brain processing. Psychol. Bull. 135, 731–748. 10.1037/a0016570, PMID: 19702380 PMC2890316

[ref98] Watson D. (2001). Dissociations of the night: individual differences in sleep-related experiences and their relation to dissociation and schizotypy. J. Abnorm. Psychol. 110, 526–535. 10.1037/0021-843X.110.4.526, PMID: 11727942

[ref99] Watson D. Stasik S. M. Ellickson-Larew S. Stanton K. (2015). Explicating the psychopathological correlates of anomalous sleep experiences. Psychol. Conscious. Theory Res. Pract. 2, 57–78. 10.1037/cns0000038

[ref100] Westen D. Weinberger J. Bradley R. (2007). “Motivation, decision making and consciousness: from psychodynamics to sublimal priming and emotional constraint satisfaction” in The Cambridge handbook of consciousness. eds. Zelazo P. D. Moscovitch M. Thompson E. (New York: Cambridge University Press), 673–702.

[ref101] WHO (2010). International Classification of Diseases [Online]. Available at: http://www.who.int/classifications/icd/en/ (Accessed February 9, 2012).

[ref102] Zelazo P. D. Gao H. H. Todd R. (2007). “The development of consciousness” in The Cambridge handbook of consciousness. eds. Zelazo P. D. Moscovitch M. Thompson E. (New York: Cambridge University Press), 405–432.

